# As a biomarker for gastric cancer, circPTPN22 regulates the progression of gastric cancer through the EMT pathway

**DOI:** 10.1186/s12935-020-01701-1

**Published:** 2021-01-11

**Authors:** Shuo Ma, Shan Kong, Xinliang Gu, Yanhua Xu, Mei Tao, Lei Shen, Xianjuan Shen, Shaoqing Ju

**Affiliations:** 1 Department of Laboratory Medicine, Affiliated Hospital of Nantong University, Xisi Road, No. 20, Nantong, 226001 Jiangsu China; 2 Research Center of Clinical Medicine, Affiliated Hospital of Nantong University, Nantong, 226001 Jiangsu China; 3 Medical School of Nantong University, Nantong University, Nantong, 226001 Jiangsu China

**Keywords:** Gastric cancer, circPTPN22, Diagnosis and prognosis, Biomarker, Epithelial-mesenchymal transformation

## Abstract

**Background:**

Gastric cancer (GC) is one of the most common cancers in the world. Due to the lack of specific symptoms, more than 80% of patients are diagnosed as the advanced stage with a high mortality rate, so the early diagnosis of GC is incredibly essential. Circular RNAs (CircRNAs) are a kind of endogenous non-coding RNA with stable structure, the long half-life, and tumor specificity. It can be used as a diagnostic marker for tumors.

**Method:**

Using circRNA sequencing technology screened three pairs of GC and adjacent tissues, and circRNAs with significant expression differences were screened out. The circular structure and characteristics of circPTPN22 were determined by RT-qPCR, agarose gel electrophoresis, Sanger sequencing, RNase R, and actinomycin D assays. Cell Counting Kit‐8, colony formation, Transwell, Wound healing, tumor formation in mice and western blotting assays were used to detect the effects of circPTPN22 on the proliferation, invasion, migration, tumor growth of GC cells in vitro and protein expression.

**Result:**

CircPTPN22 is up-regulated and positively correlated with metastasis in GC tissues, cells, and plasma. RT-qPCR results showed that circPTPN22 had good diagnostic efficacy and could be used to predict the prognosis of GC patients. In vitro and vivo experiments showed that the downregulation of circPTPN22 could inhibit cell proliferation, migration, and invasion through the epithelial-mesenchymal transformation (EMT) pathway. CircPTPN22 may regulate GC progression through the competitive binding of miRNAs.

**Conclusion:**

CircPTPN22 can be used as a potential diagnostic and prognostic marker for GC and can inhibit cell proliferation and metastasis through the competitive binding of miRNA to inhibit the EMT pathway.

## Introduction

Gastric cancer (GC) is one of the most common cancers globally, and the third-largest cause of cancer-related deaths after lung and liver cancer [1]. Although the development of surgical treatment and adjuvant therapy has significantly improved the prognosis of GC patients in recent years, the overall survival rate is still unsatisfactory, especially in advanced tumors where distal metastasis is common. This phenomenon is mainly due to its heterogeneity and complex regulatory relationships at the molecular level [2, 3]. Therefore, the continued exploration and discovery of new biomarkers with strong sensitivity and specificity is of great clinical significance for the early diagnosis and prognosis evaluation of GC.

CircRNAs are formed by splicing events after exon or intron cyclization [4, 5], and their expression abundance is high. About one-eighth of the genes expressed in humans can produce detectable circRNAs, and their expression level is more than ten times of the corresponding linear messenger RNA (mRNA) level [4, 6]. In general, circRNAs are specifically expressed in different tissues and at different developmental stages [7]. Meanwhile, it is relatively stable and resistant to RNA exonuclease or Ribonuclease R (RNase R) [8]. With the development of research, circRNAs have been considered as microRNA (miRNA) sponge, protein bait, RNA splicing regulator, parent gene transcription regulator, and potential protein translation template [9, 10]. CircRNAs have demonstrated advantages as biomarkers for tumor diagnosis and treatment due to its biological properties and functions. Besides, a growing body of evidence indicates that circRNAs is closely related to human diseases, especially cancer.

Metastasis is an essential factor in the behavior of malignant tumors. Many studies have shown that circRNAs can regulate tumor progression through the epithelial-mesenchymal transformation (EMT) pathway. For example, circHMCU is up-regulated in cell lines with high metastatic potential for breast cancer. In vitro and in vivo experiments have shown that circHMCU significantly promotes the proliferation, migration, and invasion of breast cancer cells through the EMT pathway. It can be used as a biomarker for early risk assessment, clinical treatment, and survival assessment [11]. Hsa_circ_0000177 can promote migration and invasion of glioma cells by activating the Wnt/β-catenin pathway and enhancing the EMT pathway [12]. CircPTK2 up-regulated in colorectal cancer tissues can promote EMT process of colorectal cancer cells in vitro and in vivo by binding to vimentin at Ser38, Ser55, and Ser82 sites. It plays an important role in the growth and metastasis of colorectal cancer and maybe a potential target for the treatment of colorectal cancer metastasis, as well as a promising biomarker for the early diagnosis of metastasis [13]. These studies indicated that EMT is an important pathway for tumor metastasis, and the expression level of circRNAs is strictly controlled according to different environments [14]. The exact mechanism by which circRNA regulates EMT is unclear. CircRNA-based diagnosis and treatment face many challenges.

Different from other biomarkers of GC, circPTPN22 was up-regulated in GC tissues, plasma and cells, and was positively correlated with metastasis. CircPTPN22 had a positive diagnostic effect and could track the postoperative recovery of patients. Through in vivo and in vitro experiments, we verified that the down-regulation of circPTPN22 effectively inhibited the proliferation and metastasis of GC cells through the EMT pathway. It was also predicted that it might act as ceRNA binding to has_miR_665 to regulate the development of GC.

## Methods

### Human samples

120 plasma of GC patients, 104 plasma of healthy donors, and 70 plasma of gastritis patients were obtained from the Clinical Laboratory, Affiliated Hospital of Nantong University. In this study, all patients were clinically diagnosed with GC and gastritis without any preoperative treatment. Forty pairs of GC tissue specimens and adjacent cancer specimens were from the Department of Pathology of Affiliated Hospital of Nantong University. All the tissue samples were diagnosed as gastric cancer by pathologists. Immediately after tissue excision, the tissue was stored in an RNA fixator Bioteke (Nantong, China) and placed in a − 80 °C refrigerator. Specimens were collected from September 2015 to January 2020. The ethics committee of the local hospital (ethical review report number: 2018-L055) approved the study. All participants obtained informed consent before the clinical trial and gave consent to publish.

### CircRNA sequencing

We extracted total RNA from GC tissue samples. We evaluated the concentration and integrity of the total RNA with Qubit 3.0 Fluorometer (Invitrogen, Carlsbad, California) and Agilent 2100 Bioanalyzer (Applied Biosystems, Carlsbad, CA). The extracted RNAs were purified to remove linear RNA by digestion with RNase R after removing ribosomal RNA (Geneseed, Guangzhou, China). KAPA RNA HyperPrep Kit with RiboErase (HMR) for Illumina (Kapa Biosystems, Inc., Woburn, MA) was used to prepare sequencing libraries. CircRNA Sequencing analysis was performed by Illumina Hiseq Xten via PE150 sequencing mode.

### Total RNA and gDNA extraction and RT-qPCR

Total RNA in GC tissues, cells and plasma were extracted by TRIzol Reagent and TRIzol LS reagent (Invitrogen, Canada). According to the Revert Aid First Strand cDNA Synthesis Kit (Thermo Fisher Scientific, MA, USA), genomic DNA (gDNA) is extracted from tissues and cells. The total RNA was reverse-transcribed into cDNA using a reverse transcription kit (Thermo Fisher Scientific, MA, USA). The specific divergent primers (Ribobio) were designed, with the forward and reverse primer as “5′-TCACAAGGAGTAAGGAGAAT-3′” and “5′-CTCTTGCTTGGTCTAAGTATC-3′”. Roche 480 real-time fluorescence quantitative PCR was used to amplify the 20ul system. According to the relative quantitative analysis of the 2^−ΔΔCT^ method. PCR products were further identified by Sanger sequencing.

### RNase R and actinomycin D assays

5ul total RNA was treated with RNase R (Geneseed, China). The amount of RNase R needs to be calculated based on the total RNA. The 20ul reaction system was used to incubate the RNase R at 37 °C for 2 min, and the RNase R was inactivated at 70 °C for 10 min, after which the RNase R was reversely transcribed into Complementary DNA (cDNA) for backup use. The actinomycin D of 1 mg/ml was diluted to 2.5 μg/ml using RPMI-1640 medium, and the ordinary medium was replaced in the 6-well plate. After treatment, total RNA was extracted from the cells for subsequent determination at 0, 2, 4, 8, 12, 24 h, respectively.

### Cell culture

GC cells (MKN-1, MKN-45, BGC-823, MGC-803, SGC-7901, HGC-27, AGS) and GASTRIC epithelial cells (GES-1) were obtained from the Chinese Academy of Sciences (Shanghai, China). The cells were cultured in RPMI-1640 medium (Corning, USA) + 10% Fetal bovine serum (FBS) (Gibco, USA) and 1% tri antibody (Sheng Gong, Nantong, China). Culture at 37 °C, 5%CO_2_. Change the liquid every 2 days.

### Plasmid construction and cell transfection

Build circRNA shRNA carrier through online software to predict two shRNA fragments, with interval sequence fragment sequence of forward and reverse links interference, plus enzyme loci on both ends, with the hairpin shRNA interference fragment, the annealing product access pGPU6/GFP/Neo carrier, heat into e. coli DH5a, 37 °C in tablet containing streptomycin training for the night, choose positive colony amplification extracted plasmid. The sh1 and sh2 plasmid sequences are “5′-GAGTAAGGAGAATTCTCACCA-3′” and “5′-CACAAGGAGTAAGGAGAATTC-3′”. After the cells were transferred to the third generation and fusion to 70~ 80%, they were inoculated in 6-well plates. Plasmids containing sh-control, sh-1, and sh-2 were mixed with lipo3000 (Thermo Fisher Scientific, MA, USA) and incubated for 10 min. During the incubation period, the cells were exchanged for liquid, and lipo containing plasmids were added into the newly changed medium.

### CCK8 and clone formation assays

Two days after cell transfection, 3000 cells per well were counted and inoculated uniformly in the 96-well plate. After the cells were adherent to the wall, 10ul cck8 reagent (Dojindo, Kumamoto, Japan) was added. After incubation for 2 h, the absorbance of the assay Wells at 450 nm and 630 nm was measured using a Microplate Reader. In the cell cloning and formation assay, the transfected cells were counted at 1000 cells per well and inoculated uniformly in the 6-well plate. The liquid was changed every 4 days to ensure the normal growth of cells for 2 weeks. 1 ml 4% paraformaldehyde was used for fixation for 12 h. Crystal violet was dyed for 10 min and then washed with PBS and photographed.

### Wound healing assay

Transferred to the third generation, the cells were inoculated in a 6-well plate, and the sterile pipet-tip was evenly scratched along the center of the hole after 8–12 h of wall fixation. After that, the culture medium was replaced with the transfected plasmid, and images were taken immediately (0 h) under an inverted microscope and labeled to facilitate the location of the visual field. The culture at 37 °C and 5% CO_2_ for 24 h, the culture bottle was taken out, and images were taken in the same field after rinsed with PBS.

### Transwell assay

Transwell assay included migration and invasion assays. Cells were digested down within 24–48 h after transfection and counted according to 50,000 cells per migration well and 80,000 cells per invasion well. The migrating cells were uniformly inoculated in a 24-well plate, and the invading cells were uniformly inoculated in a 24-well plate containing matrix glue, which was shaken well and then cultured in an environment of 37 °C and 5% CO_2_. After 48 h, 4% paraformaldehyde was used for fixation, migration and fixation for 30 min, and invasion and fixation for 12 h. After the end, crystal violet was dyed for 10 min and photographed under the microscope.

### Tumor formation assay in nude mice

GC cells (1*10^7^) were inoculated subcutaneously in female nude mice. From the day of inoculation, tumor length (a) and short diameter (b) were measured every 2 days and tumor volume (V = a*b^2^/2) was calculated to draw the tumor growth curve. After 30 days, the nude mice were sacrificed and the tumor was completely exfoliated. The tumor size was measured and weighed. RT-qPCR was used to verify the expression of circPTPN22, E-cadherin (E-cad) and Vimentin in tumor tissues, The protein content of E-cad and Vimentin in tumor tissues was detected by Western blots.

### Western blots

Cells were lysed with RIPA buffer and PMSF (Solarbio, China), separated by 10% SDS-PAGE (Epizyme Biotechnology, Shanghai, China), and electro-blotted onto a PVDF membrane (Millipore). After blocking with 5% nonfat milk, the membrane was incubated with various specific primary antibodies (CST) overnight at 4 °C. Blots were washed and incubated with horseradish peroxydase-coupled secondary antibodies (CST) for 2 h. The protein bands were detected using the Pro-lighting HRP agent. Expression of β-actin was used as a loading control.

### Statistical analysis

Data from three independent assays were denoted as mean ± SD. Statistical analysis was conducted using the Statistical Product and Service Solutions (SPSS) (SPSS Inc., Chicago, IL, USA) and GraphPad Prism 8 software (GraphPad Software, San Diego, CA). The significance of the variance was evaluated by Student’s t-test or ANOVA. Overall survival of GC patients by Kaplan–Meier analysis and Pearson’s correlation analysis found the correlation. P < 0.05 has statistical significance.

## Result

### The expression profiles of circRNAs in GC and the screening of circPTPN22

**Fig. 1:**
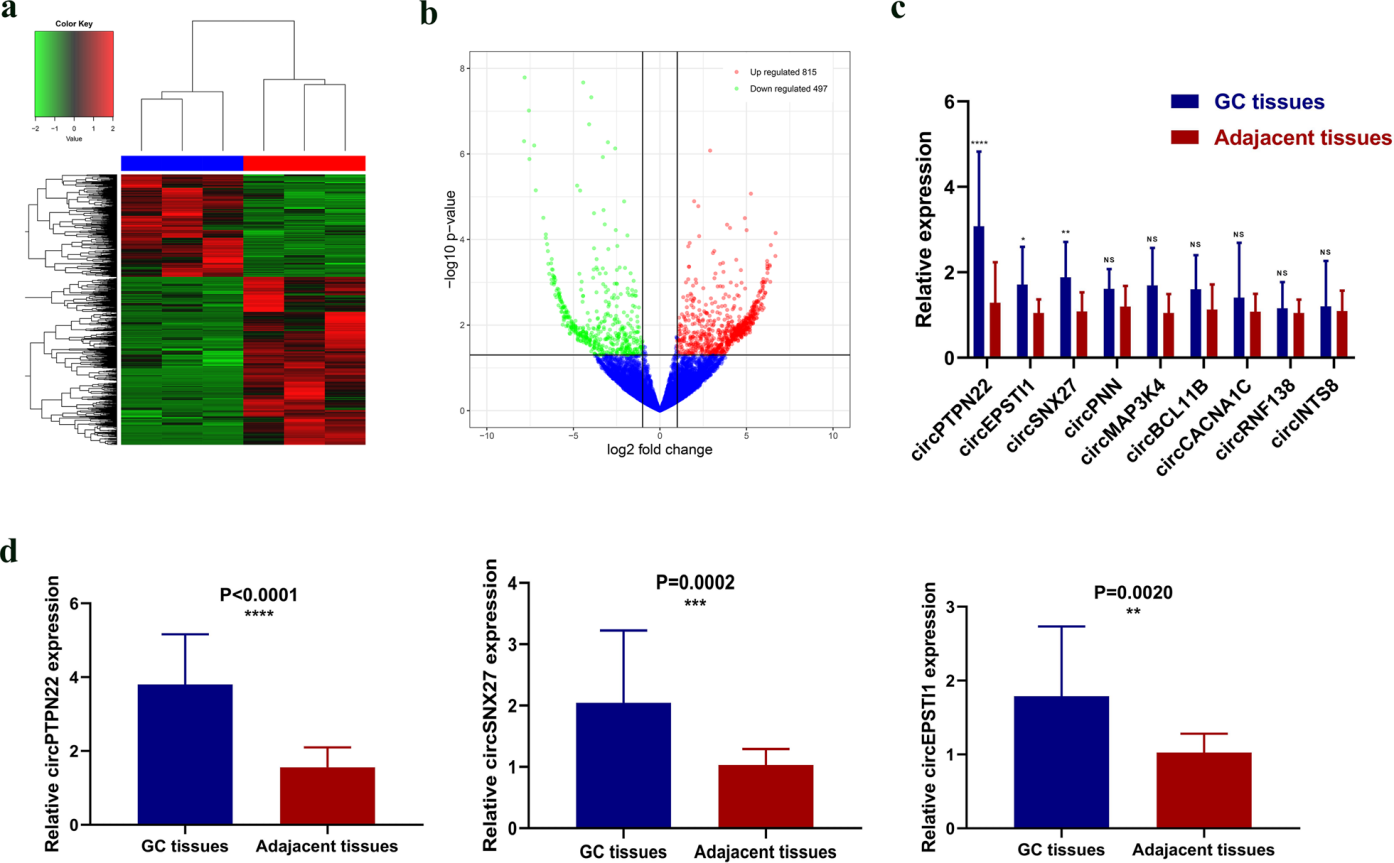
Identification and screening of circPTPN22. **a**, **b** CircRNA sequencing was performed on 3 pairs of GC tissues and adjacent non-cancer tissues. **c** Expression levels of 9 circRNAs in GC tissues. **d** The expression levels of circPTPN22, circSNX27, and circEPSTI1 in metastatic and non-metastatic GC tissues

To identify the differentially expressed circRNAs in GC tissues, we used Circular RNA Sequencing to analyze 3 pairs of GC tissues and their matched adjacent non-tumor tissues. We visualized the distinguishable expression patterns between the control and experimental groups using heat maps (Fig. 1a) and analyzed the differences in the expression levels of different circRNAs in the tissue using volcanic maps (Fig. 1b). Nine up-regulated circRNAs (circRNAs with a fold change > 2 and a P-value < 0.05) were found in GC tissues, and the information was shown in Table 1. Subsequently, RT-qPCR assay was used to verify the difference in expression levels of these 9 circRNAs between 20 pairs of GC tissues and their matched adjacent non-tumor tissues. It was found that the expression levels of 3 circRNAs (circPTPN22, circSNX27, circEPSTI1) were significantly up-regulated as compared with the sequencing results. However, circPNN, circMAP3K4, circBCL11B, circRNF138, circCACNA1C, and circINTS8 were not differentially expressed in GC tissues and their matched adjacent non-tumor tissues (Fig. 1c). In addition, to determine the circRNA with the highest correlation with GC metastasis, RT-qPCR was used to verify the difference of expression levels of the three circRNAs in 20 pairs of GC metastatic tissues and their adjacent non-tumor tissues, the results show that circPTPN22 (also known as hsa_circ_0000110) showed the most obvious difference in expression and the highest expression in the metastatic GC specimens (Fig. 1d). Subsequently, we conducted in-depth research on it.

**Table 1:** Basic information of circRNAs after sequencing

circRNA	circBase ID	Gene	Position	Length
circEPSTI1	hsa_circ_0000479	EPSTI1	chr13: 43528083–43544806	375
circSNX27	hsa_circ_0000130	SNX27	chr1: 151611363–151611595	232
circRNF138	hsa_circ_0005729	RNF138	chr18: 29691716–29693823	282
circPNN	hsa_circ_0101802	PNN	chr14: 39648294–39648666	295
circMAP3K4	hsa_circ_0078619	MAP3K4	chr6: 161469647–161471011	1364
circBCL11B	hsa_circ_0103051	BCL11B	chr14: 99723807–99724173	366
circCACNA1C	hsa_circ_0025016	CACNA1C	chr12: 2224389–2229596	428
circINTS8	hsa_circ_0001814	INTS8	chr8: 95839490–95854716	955
circPTPN22	has_circ_0000110	PTPN22	chr1: 114372213–114377061	356

### Basic information and characteristics of circPTPN22

**Fig. 2:**
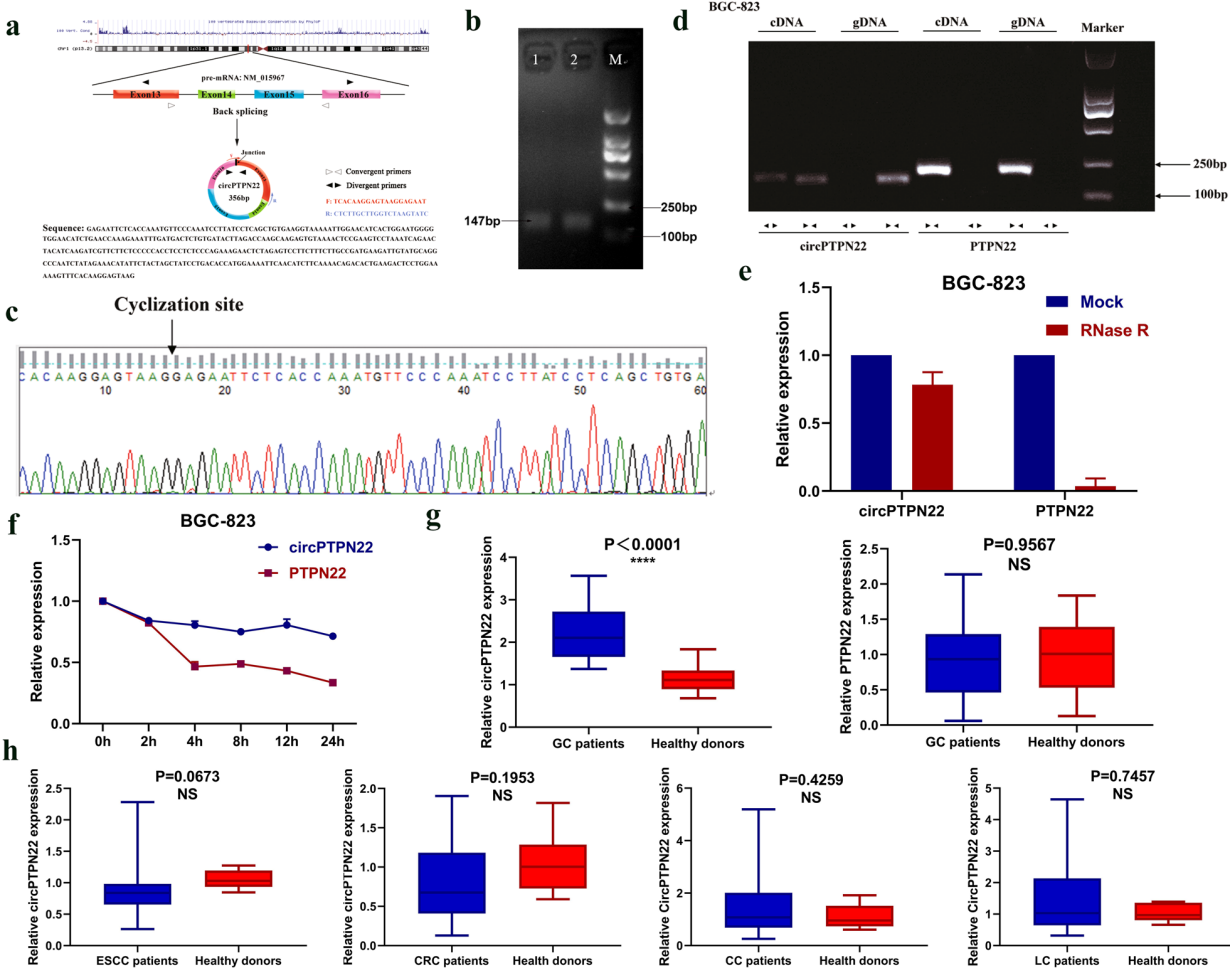
Basic information and characteristics of circPTPN22. **a** Location and formation of circPTPN22. **b** The primer length of circPTPN22 was verified by agarose gel electrophoresis. **c** Sanger sequencing detects the cyclization sites. **d** Verify circPTPN22 circular structure. **e** RNase R assay verified the tolerance of circPTPN22 to exonuclease. **f** Actinomycin D assay verified the half-life of circPTPN22 in BGC-823 cell. **g** The expression levels of circPTPN22 and its transcription gene PTPN22 in plasma of 20 GC patients and 20 healthy donors. **h** The expression levels of circPTPN22 in plasma of 20 ESCC, CRC, CC, LC patients and 20 healthy donors

According to the circBase database (http://www.circbase.org/), it was found that circPTPN22 was located in chr1:114,372,213-114,377,061, and the mature transcript length of circPTPN22 was 356 bp (Fig. 2a), and circPTPN22 was composed of exons 13, 14, 15, and 16 by the circPrimer software analysis (Fig. 2a). As is known to all, circRNAs generally form closed rings by back splicing, so we designed a diverging primer (Fig. 2a) capable of reverse amplification of the circular molecule according to the circPTPN22 cyclization site. Then, the expression levels of circPTPN22 were detected by RT-qPCR, and the PCR amplification products were detected by 3% agarose gel electrophoresis. The electrophoresis band was 147 bp, which was consistent with the size of the primer amplification products (Fig. 2b). The reverse shear site of circPTPN22 was further confirmed by Sanger sequencing (Fig. 2c). In addition, agarose gel electrophoresis was performed on the products of genomic DNA (gDNA) and complementary DNA (cDNA) PCR to verify the cyclogenesis of circPTPN22 and confirm its circular structure (Fig. 2d). Many nucleases in the cells can easily degrade RNA molecules, while circRNA has better stability and is not easily degraded. Therefore, we treated BGC-823 cells with RNase R and found that circPTPN22 was not easily degraded, while its transcription gene PTPN22 was significantly degraded (Fig. 2e). In addition, since actinomycin D can inhibit RNA production by inhibiting RNA polymerase, BGC-823 cells were cultured in the medium containing actinomycin D for 24 h. RT-qPCR showed that the half-life of circPTPN22 was significantly higher than that of its transcription gene PTPN22, which further demonstrated the stability of circPTPN22 (Fig. 2f). Meanwhile, we detected the expression levels of circPTPN22 and its transcription gene PTPN22 using plasma from 20 GC patients and 20 healthy donors and found that only circPTPN22 had significant expression differences (P < 0.0001), while the transcription gene PTPN22 was not significantly different (P = 0.9567) (Fig. 2g). To determine whether circPTPN22 is specifically expressed only in GC, we select 20 patients plasma with esophageal squamous cell carcinoma (ESCC), colorectal cancer (CRC), cervical cancer (CC), and lung cancer (LC) and 20 cases of healthy donors, respectively plasma is used to detect the expression levels, found the expression levels of circPTPN22 differences had no statistical significance in ESCC, CRC, CC, and LC (The P values are 0.0673, 0.1953, 0.4259, 0.7457, respectively). The above experiments suggested that the expression correlation of circPTPN22 in the gastrointestinal system was higher than that in the non-gastrointestinal system and was only differentially expressed in GC, indicating the specificity of circPTPN22 expression levels in the diagnosis of GC (Fig. 2h).

### Verification of clinical trial methods and diagnostic significance of circPTPN22 in GC plasma

To explore whether the detection of circPTPN22 can be applied in clinical practice, we first conducted a comprehensive evaluation of its detection methods. We used mixed plasma to determine the precision of circPTPN22 and found that the inter-assay coefficient of variation (CV) and intra-assay CV performed well (Additional file 1: Table S1). Subsequently, when mixed plasma was placed at room temperature for 0, 6, 12, 18, 24 h and repeated freezing and thawing for 0, 1, 3, 5, and 10 times (Additional file 2: Fig. S1a, b), no significant difference was found in the expression levels of circPTPN22, indicating that the detection of circPTPN22 is not affected by this factor and has good stability (P < 0.05). In addition, we used a tenfold diluted circPTPN22 standard recombinant plasmin for linear evaluation, with a standard curve R2 of 0.9996 and a regression equation of Y = −3.288x + 35.429, suggesting that quantitative reverse transcription-polymerase chain reaction can be used to detect circPTPN22 in plasma at different concentrations (Additional file 2: Fig. S1c). A smooth, single-peak fusion curve can also demonstrate the specificity of the method (Additional file 2: Fig. S1d). In conclusion, the clinical feasibility of this method was confirmed.

**Fig. 3:**
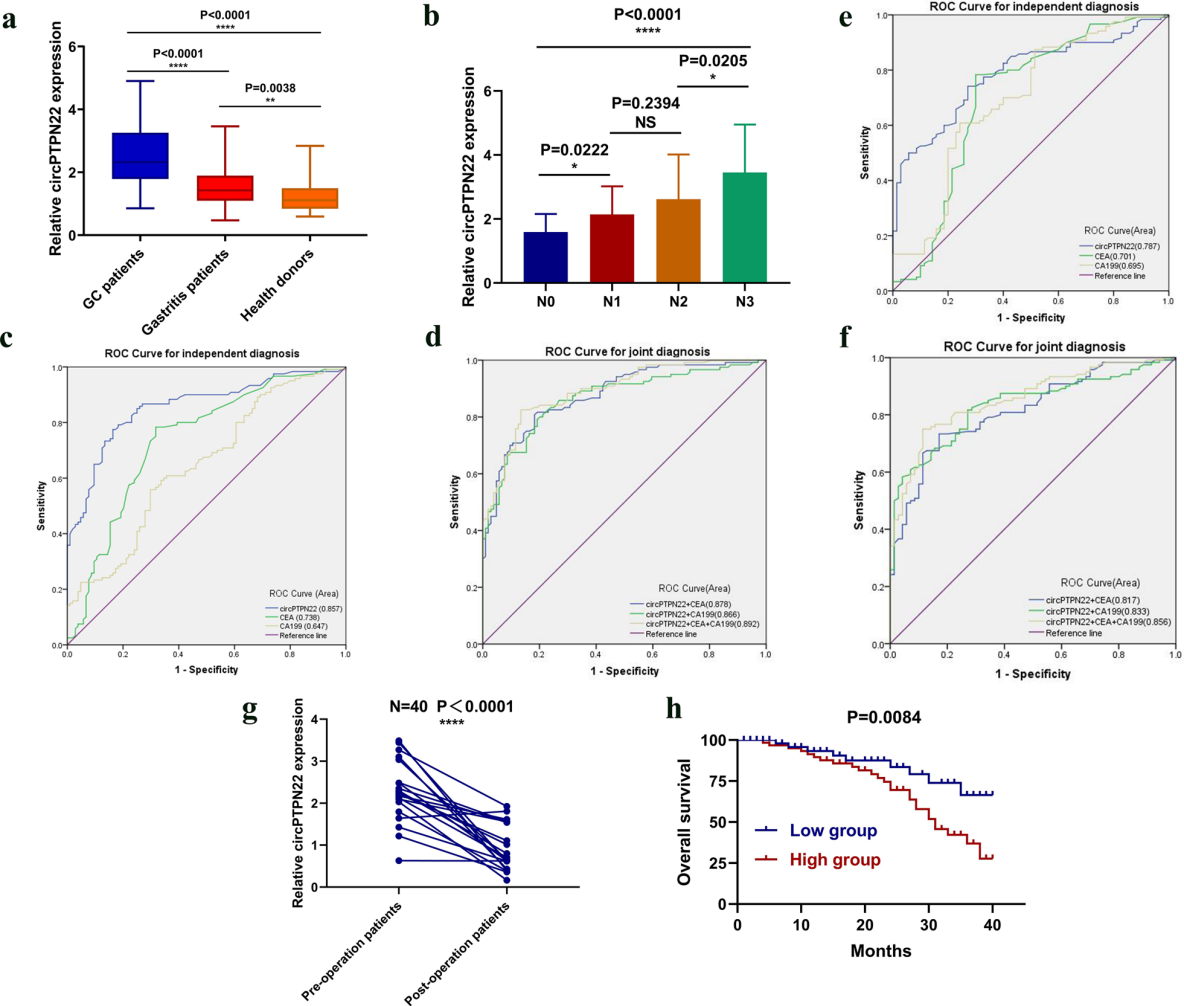
The diagnostic and prognostic value of circPTPN22 in GC. **a** The expression levels of circPTPN22 in plasma specimens of GC primary patients (n = 120), gastritis patients (n = 70), and healthy donors (n = 104). **b** The expression levels of circPTPN22 in plasma specimens of GC at different stages of lymph node metastasis. **c**, **d** ROC curve analysis of circPTPN22, CEA, and CA199 in independent diagnosis and joint diagnosis of GC patients and healthy donors. **e**, **f** ROC curve analysis of circPTPN22, CEA, and CA199 in independent diagnosis and joint diagnosis of GC patients and gastritis patients. **g** The expression levels of plasma circPTPN22 in the 20 GC patients before and after surgery. **h** Kaplan–Meier survival curve verifies the prognostic value of circPTPN22

To investigate the diagnostic significance of circPTPN22 in GC plasma, plasma samples from 120 patients with GC, 70 patients with gastritis, and 104 healthy donors were collected to detect the difference in expression levels. We found that the expression levels of circPTPN22 showed a gradient relationship under three conditions. The plasma expression level of gastritis patients was higher than that of the healthy donor group but significantly lower than that of GC patients. In addition, the expression level in plasma of GC patients was higher than that of healthy donors (Fig. 3a). In order to explore whether circPTPN22 can also show the difference in metastasis in plasma, we compared the plasma expression level of circPTPN22 in 120 GC patients at different stages of lymph node metastasis, and the results showed that the expression level increased gradually with the deepening of lymph node metastasis (Fig. 3b). This indicated that circPTPN22 expression level was positively correlated with the degree of metastasis, except for upregulation in GC. It is well known that Carcinoembryonic antigen (CEA) and Carbohydrate antigen199 (CA199) are commonly used auxiliary diagnostic markers for gastric cancer. Therefore, we used 120 patients with GC and 104 healthy donors to conduct the Receiver operating characteristic (ROC) analysis of circPTPN22 and CEA and CA199 to determine the diagnostic effect of circPTPN22 in GC plasma. The ROC curve showed that the AUC value of circPTPN22 is 0.857 (95% confidence interval is 0.808–0.907, P < 0.001). Its AUC value is higher than CEA (0.738, 95% confidence interval: 0.671–0.804, P < 0.001) and CA199 (0.647, 95% confidence interval: 0.575–0.719, P < 0.001) (Fig. 3c). In addition, circPTPN22 is higher than CEA and CA199 in terms of sensitivity (78%), specificity (84%), overall accuracy (80%), positive predictive value (84%), and negative predictive value (76%) (Table 2). Then, combined ROC analysis was performed on the circPTPN22, and it was found that the specificity increased sensitivity (83%) after combined use. The AUC value of circPTPN22 is 0.878 after combined use with CEA, 0.866 after combined use with CA199, and the maximum AUC value of circPTPN22 is 0.892 (Fig. 3d). The above analysis showed that combined use could make up for the limitations of a single marker, and circPTPN22 as a biomarker has potential in the diagnosis of GC. It is particularly important for us to distinguish GC from gastritis because the early diagnosis of many GC patients is not easily discovered, and quite a few of them are transformed by gastritis. ROC curve analysis showed that the AUC value of circPTPN22 (0.787, 95% confidence interval: 0.723–0.851, P < 0.001) is higher than CEA (0.701, 95% confidence interval: 0.616–0.786, P < 0.001) and CA199 (0.695, 95% confidence interval: 0.614–0.776, P < 0.001) (Fig. 3e) in distinguishing GC and gastritis, and the sensitivity was also significantly increased after combined diagnosis, with the AUC value of circPTPN22 is 0.817 after combined use with CEA, 0.833 after combined use with CA199, and The AUC value of the three is 0.856 after combined use (Fig. 3f). Therefore, circPTPN22 can be used as a marker to distinguish between gastric cancer and gastritis.

**Table 2:** Use the expression levels of circPTPN22, CEA, and CA199 to distinguish gastric cancer patients from healthy donors

	SEN, %	SPE, %	ACCU, %	PPV, %	NPV, %
CircPTPN22	0.78 (93/120)	0.84 (87/104)	0.80 (180/224)	0.85 (93/110)	0.76 (87/114)
CEA	0.75 (90/120)	0.68 (71/104)	0.72 (161/224)	0.73 (90/123)	0.70 (71/101)
CA199	0.71 (85/120)	0.40 (42/104)	0.57 (127/224)	0.58 (85/147)	0.55 (42/77)
CircPTPN22 + CEA	0.82 (98/120)	0.81 (84/104)	0.81 (182/224)	0.83 (98/118)	0.72 (84/116)
CircPTPN22 + CEA + CA199	0.83 (99/120)	0.87 (90/104)	0.84 (189/224)	0.88 (99/113)	0.81 (90/111)

### Clinical value of circPTPN22 in GC plasma, real-time monitoring ability and prognostic value of GC patients

To determine the clinical application value of circPTPN22, we collected and analyzed clinicopathological data of 120 patients with GC (Table 3). 120 patients with GC were divided into a relatively high expression group (expression level > 2.25, n = 60) and a relatively low expression group (expression level ≤ 2.25, n = 60). As can be seen from the table, the expression level of circPTPN22 was positively correlated with the differentiation degree of GC (P = 0.028), Tumor size (P = 0.001), T stage (P < 0.0001), lymph node metastasis (P < 0.0001), and TNM stage (P < 0.0001), but had no statistical significance with other factors (gender, age, nerve/vascular invasion). To confirm whether circPTPN22 can be used as a key prognostic molecule, we followed the expression levels of circPTPN22 in 20 patients who were initially treated for GC. Surprisingly, the expression of circPTPN22 was significantly reduced after surgery in the same patient (Fig. 3g). The survival curve also demonstrated that the survival rate of the low-expression group was higher than that of the high-expression group (Fig. 3h). This also indicates that circPTPN22 can effectively track the postoperative situation of GC.

**Table 3:** Clinical Bentley analysis of circPTPN22

Parameter	No. of patients	circPTPN22(high)	circPTPN22(low)	P-value
Sex				
Male	72	34	38	0.456
Female	48	26	22	
Age (years)				
<60	43	18	25	0.183
≥60	77	42	35	
Tumor size				
<5	72	27	45	0.001***
≥5	48	33	15	
Differentiation grade				
Well-moderate	56	34	22	0.028*
Poor-undifferentiation	64	26	38	
T stage				
T1–T2	68	24	44	< 0.0001****
T3–T4	52	36	16	
Lymph node status				
Positive	75	24	51	< 0.0001****
Negative	45	36	9	
TNM stage				
I–II	52	15	37	< 0.0001****
III–IV	68	45	23	
Nerve/vascular invasion				
Positive	63	28	35	0.201
Negative	57	32	25	

*P < 0.05, **P < 0.01, ***P < 0.001, ****P < 0.0001

### CircPTPN22 is up-regulated and promoted cell proliferation

**Fig. 4:**
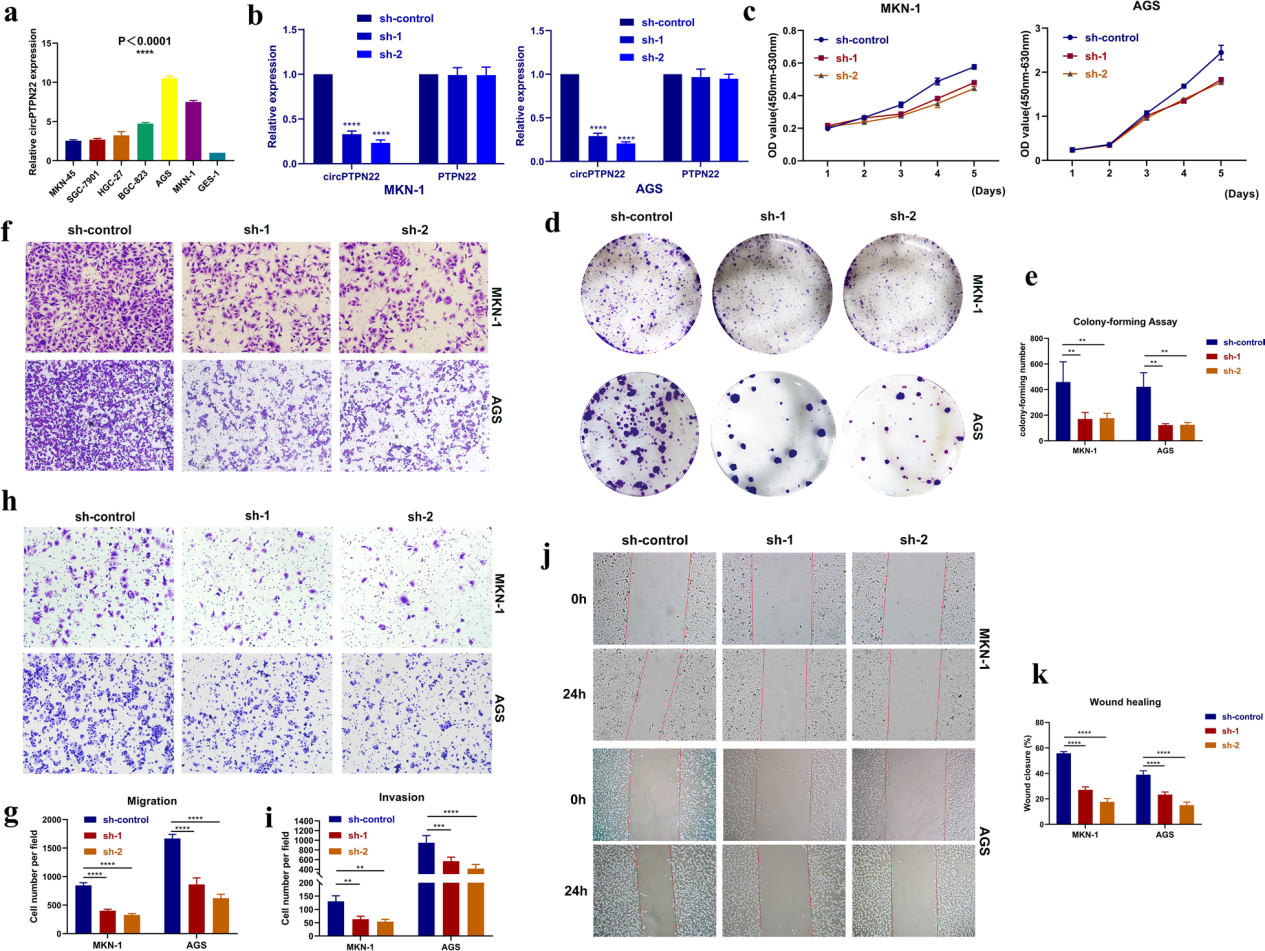
CircPTPN22 promotes proliferation and metastasis of GC cell lines. **a**  The expression levels of circPTPN22 in GC cell lines. **b** The efficiency of plasmid transfection in MKN-1 and AGS cells. **c**–**e** CCK8 assay and colony formation assay, cell proliferation was affected after circPTPN22 was knocked down. **f**–**i** Transwell assay verified the transferability after circPTPN22 was knocked down. **j**, **k** Cellular wound-healing assay was used to detect the motor ability of cells

To explore the effect of circPTPN22 on the proliferation and metastasis of GC in vitro, we conducted in vitro cell experiments. We found that circPTPN22 is up-regulated in different GC cell lines (Fig. 4a). Subsequently, knockdown plasmids were constructed to down-regulate the expression levels of circPTPN22 in GC cells, and plasmids were subsequently transfected into MKN-1 cells and AGS cells with relatively high expression of circPTPN22. Transfection efficiency was 70% ~ 80% (Additional file 3: Fig. S2a), and knockdown efficiency was 60% ~ 70% (Fig. 4b). Cck8 assay showed that the growth rate of MKN-1 and AGS cells was significantly reduced after circPTPN22 was knocked down (Fig. 4c). The colony-formation assay also proved that the cells with circPTPN22 knocked down in the same culture time had less colony formation (Fig. 4d, e). The above experiments proved that knockdown circPTPN22 could effectively inhibit cell proliferation.

### CircPTPN22 promotes cell metastasis

To investigate the effect of circPTPN22 on the metastasis ability of GC cells, we conducted Transwell assay and found that MKN-1 and AGS cells transfected with sh-1 and sh-2 significantly inhibited the migration and invasion ability of cells after 72 h of culture (Fig. 4f–i). In addition, wound healing was performed in AGS and MKN-1 cells after knockdown of circPTPN22. The scratch area was measured at 0 and 24 h after the injury, respectively. Compared with sh-control, the experimental results showed that MKN-1 and AGS cells after knockdown of circPTPN22 had a lower wound closure rate (Fig. 4j, k). The above experiments proved that knockdown of the circPTPN22 gene could inhibit the migration and invasion of GC cells and affect the metastasis ability of GC cells.

### CircPTPN22 affects the metastasis of GC cells through the EMT process

**Fig. 5:**
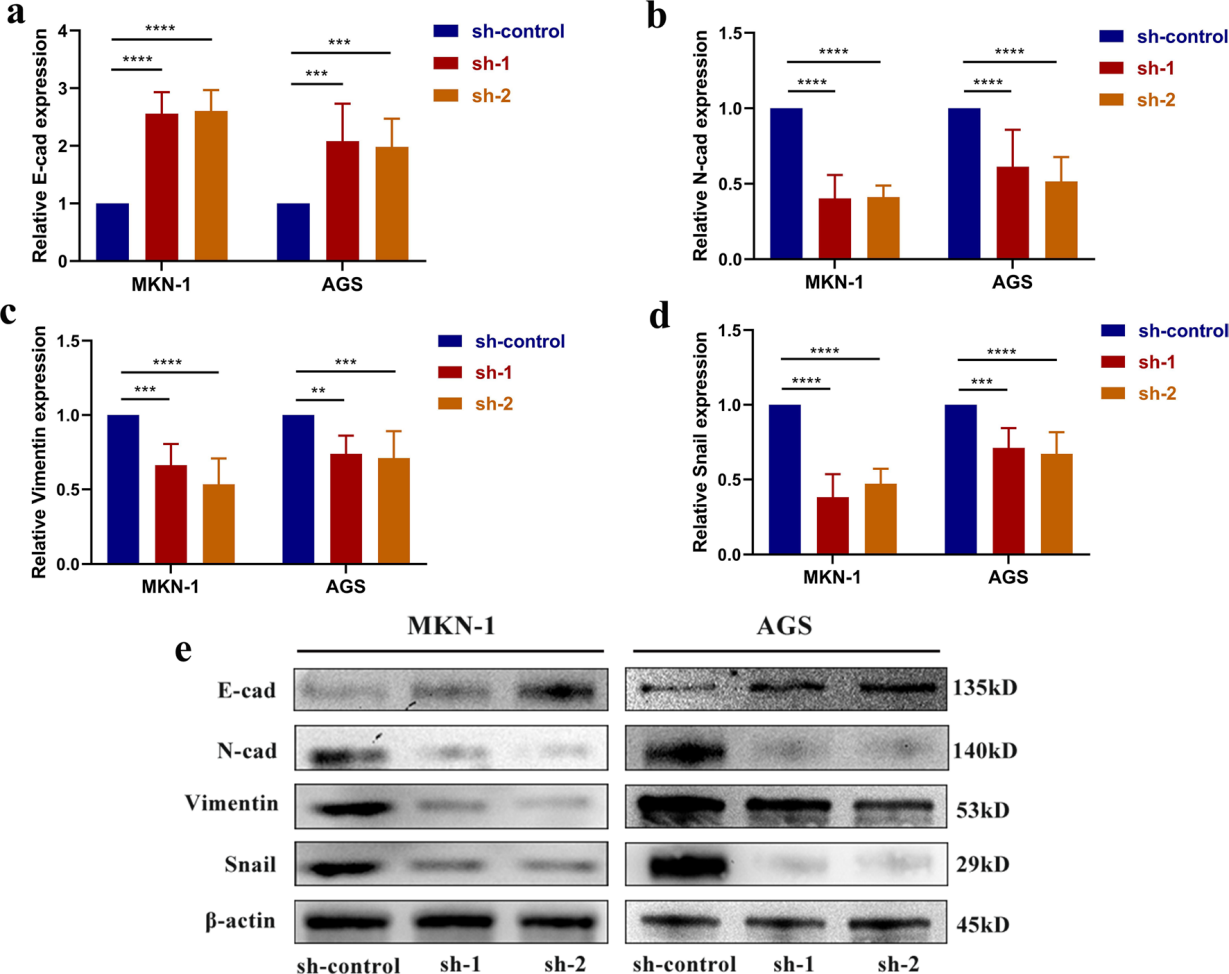
Effects of circPTPN22 on EMT process in GC cells. **a**–**d** RT-qPCR detected the effect of circPTPN22 knockdown on the expression level of key proteins (E-cad, N-cad, Vimentin, and Snail) in EMT. **e** After the downregulation of circPTPN22, major EMT proteins were expressed in MKN-1 and AGS cells

EMT accompanies most tumor cell metastasis. In most EMT processes, epithelial marker E-cad is gradually absent, while the expression levels of N-cadherin (N-cad), Vimentin, and EMT-TF factor are gradually enhanced [14]. To investigate whether circPTPN22 influences the metastasis of GC through the EMT process, we detected the expression levels of E-cad, N-cad, Vimentin, and Snail protein in GC cells of MKN-1and AGS with circPTPN22 knocked down by RT-qPCR and Western Blots. Both show the same results compared with the sh-control group, and we found that after knockdown of circPTPN22, the E-cad content in MKN-1 and AGS cells increased, while the expression levels of N-cad, Vimentin, and Snail decreased (Fig. 5a–e). The above results indicated that circPTPN22 could regulate the EMT process of GC cells and thus affect the metastasis of GC cells.

### Knockdown of circPTPN22 inhibited GC tumor growth and EMT process in vivo

**Fig. 6:**
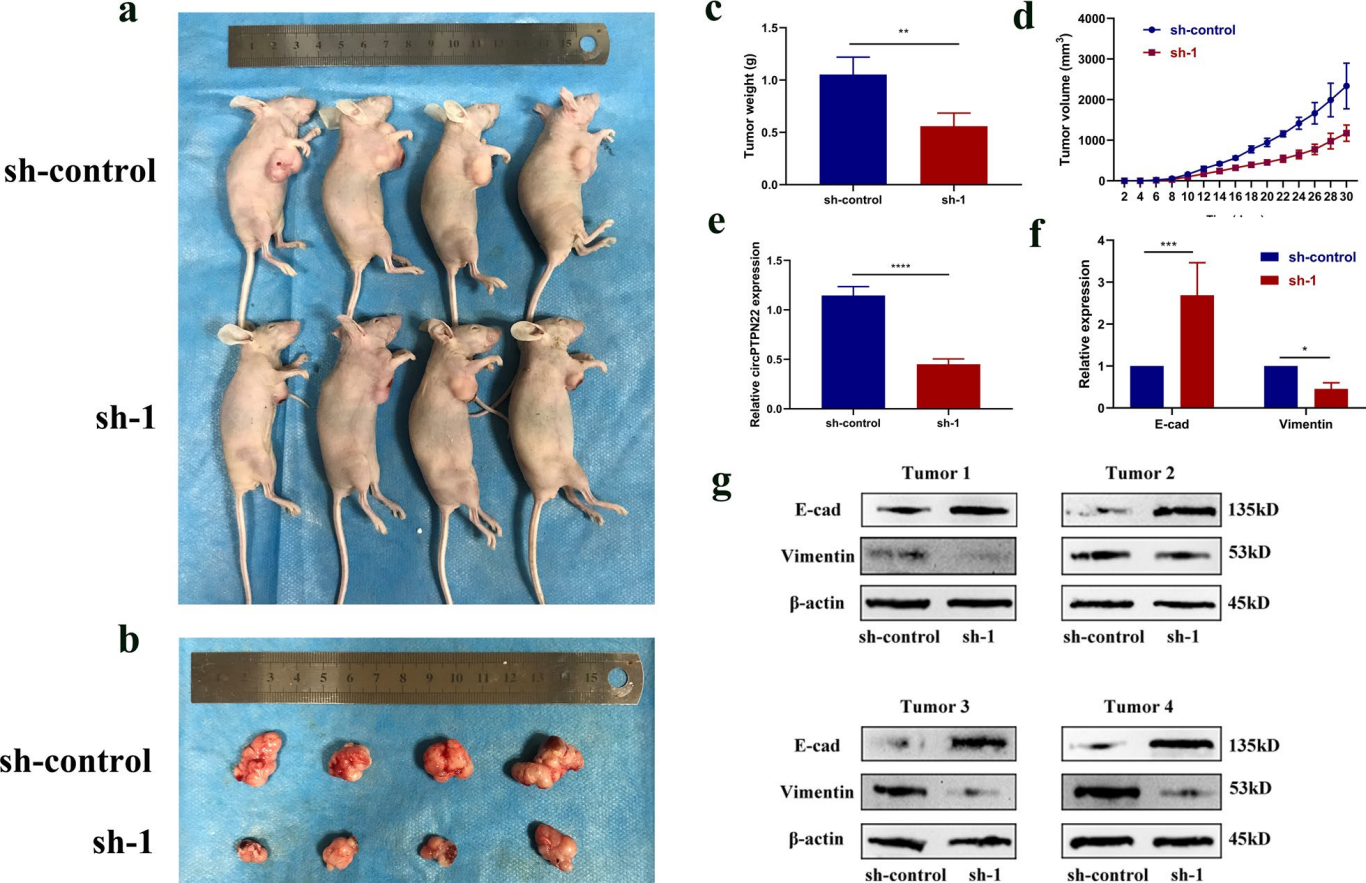
Knockdown of circPTPN22 inhibited GC tumor growth and EMT process. **a**, **b** Tumor formation model and tumor size in nude mice. **c**, **d** Compared to the sh-control group. The tumor tissue weight and volume were smaller after the downregulation of circPTPN22. **e** Expression of circPTPN22 in the tumors of the sh group and the sh-control group. **f**, **g** Expression of E-cad and Vimentin proteins in tumor in sh-group and sh-control group

To explore the effect of circPTPN22 on GC cell proliferation and EMT process in vivo, a tumorigenesis model of nude mice with GC was established, as shown in Fig. 6a and b. The downregulation of circPTPN22 resulted in tumor volume and weight smaller than those of the sh-control group. Also, compared with the sh-control group, the expression of circPTPN22 in the sh-group tumor was decreased (Fig. 6c–e). RT-qPCR results showed that after circPTPN22 was downregulated, the expression of E-cad was increased, and the expression of Vimentin was decreased. The above results were verified by Western Blots experiments (Fig. 6f and g). In summary, the downregulation of circPTPN22 can inhibit the growth of GC and EMT process in vivo.

### CircPTPN22 may act as ceRNA to regulate the progression of GC

**Fig. 7:**
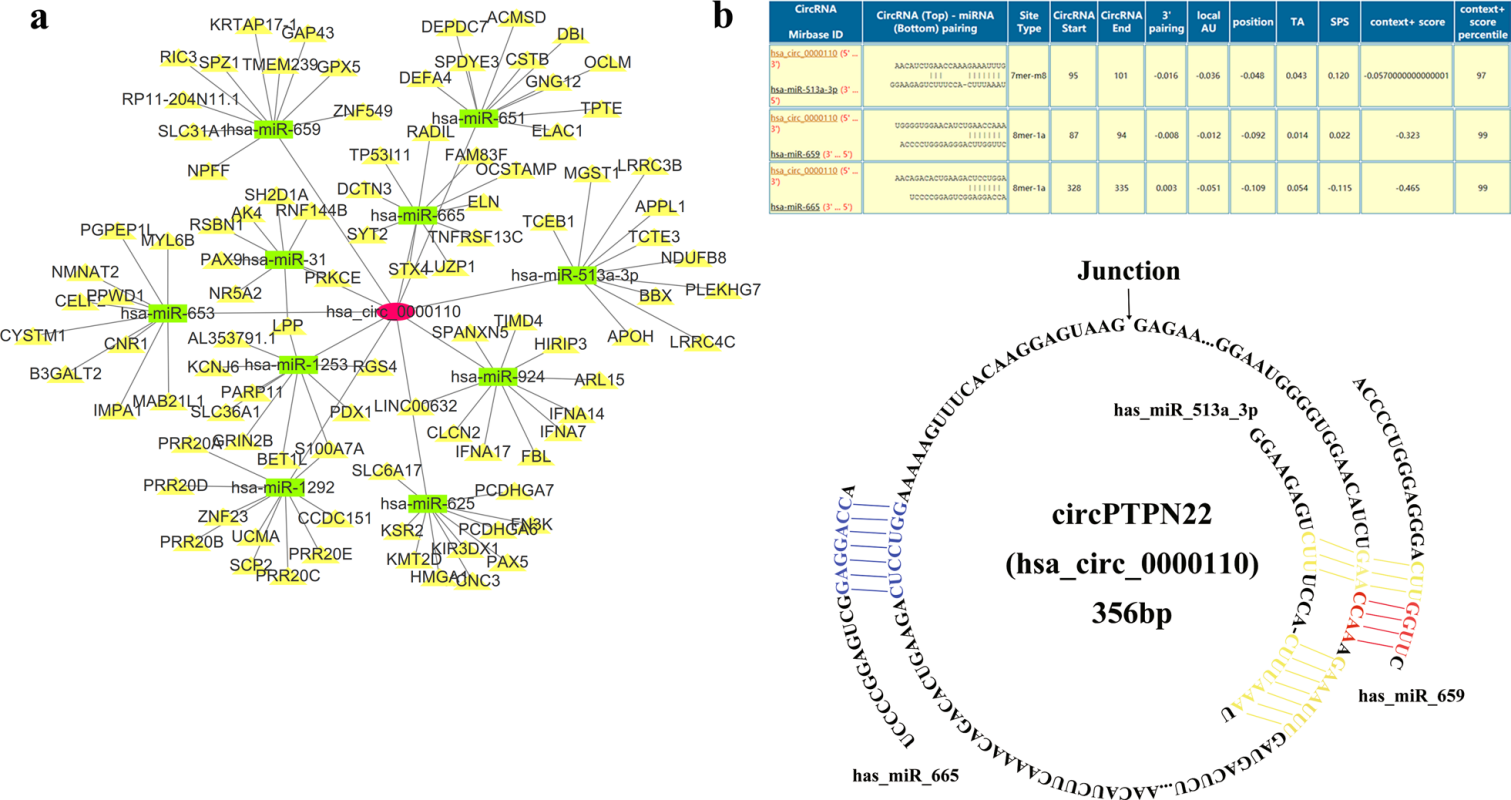
CircPTPN22 affects the mechanism of GC. **a** The network of circPTPN22 binding miRNAs. **b** Schematic diagram of circPTPN22 binding with miRNAs (has_miR_665, has_miR_659, and has_miR_513a_3p)

To explore the mechanism of circPTPN22 affecting the occurrence and development of GC, We through the circAtlas database (http://circatlas.biols.ac.cn/) to predict that circPTPN22 cannot function of encoding proteins or peptides. This led us to think of the main ceRNA mechanism of circRNA, and the circPTPN22-miRNA-mRNA axis was predicted using the circNet database (http://circnet.mbc.nctu.edu.tw/). We found that circPTPN22 connected a number of miRNAs, including has_miR_665, has_miR_659, has_miR_513a_3p, has_miR_653, has_miR_1292, has_miR_31, has_miR_1253, has_miR_625, has_miR_924, has_miR_651 (Fig. 7a). In combination with the circInteractome (https://circinteractome.nia.nih.gov/index.html) database, We selected three miRNAs of the highest score, has_miR_665, has_miR_659, has_miR_513a_3p, and produced circRNA–microRNAs connection diagram (Fig. 7b). Among them, hsa_miR_665 has been proven to regulate the metastasis of GC by affecting the EMT pathway. Has_miR_665 has proven expression in GC cells significantly down-regulated by combining PPP2R2A significant inhibition of the GC cell proliferation, invasion, and EMT process [15]. This also provides a new direction for the regulation of circPTPN22.

## Discussion

GC is the third leading cause of death worldwide, and early GC can be diagnosed with endoscopy or laparoscopy [1]. Later stages, however, usually require surgery. More than 80% of patients are diagnosed with advanced stage in the absence of specific symptoms, with a low 5-year survival rate of between 10% and 30% [15]. So, the early diagnosis of GC is particularly urgent. Recent studies have shown that circRNAs are endogenous closed non-coding RNAs (ncRNAs). It has a stable structure, long half-life, and tumor specificity [8, 16], and can be detected admire simply in various body fluids [17, 18], which provides conditions for it to be used as a tumor diagnostic marker. In addition, circRNAs can be used as the sponge of miRNAs in the occurrence and development of GC and as a diagnostic marker of GC. CircRHOBTB3 may act as ceRNA of miR-654-3p and promote GC growth inhibition by activating the p21 signaling pathway. CircRHOBTB3 may be a new diagnostic marker and therapeutic target for GC [19]. CircATXN7 promoted the development of GC by sponging miR-4319 and regulating ENTPD4, and it was found that CircATXN7 was a new biomarker in GC [20].

In our study, the differentially expressed circRNA was detected in GC tissues relative to adjacent normal GC tissues by circRNA sequencing, and the circPTPN22 was selected after the circRNA expression in metastatic and non-metastatic tissues was compared by RT-qPCR to explore whether it could be used as a marker for diagnosis and prognosis of GC and its regulatory effect. Subsequently, we confirmed that the expression level of circPTPN22 is up-regulated in GC tissues, plasma, and cells, consistent with the results of sequencing. In addition, RT-qPCR analysis showed that circPTPN22 expression was positively correlated with GC metastasis. We proved the circular structure of circPTPN22 and found that circPTPN22 was more tolerant of exonuclease and not easily degraded through experiments. The expression level of circPTPN22 in different tumors was detected. Compared with other tumors, the expression level of circPTPN22 was relatively high in gastrointestinal tumors and the highest in GC, suggesting that the expression levels of circRNA may be related to the site. All above provide evidence that circPTPN22 is an excellent diagnostic marker for GC. After ROC analysis of circPTPN22 expression in GC patients, gastritis patients, and healthy controls, it was found that its AUC was higher than that of traditional tumor markers CEA and CA199, and the AUC reached 0.892 after combined use. It can effectively distinguish GC from gastritis and GC from healthy people. In addition, follow-up and survival curves showed that circPTPN22 could serve as an independent prognostic marker. Clinicopathological data also showed that the expression level of circPTPN22 was positively correlated with tumor size, lymph node metastasis, and TNM stage in patients with GC.

In vitro experiments, downregulation of circPTPN22 can effectively inhibit cell proliferation, migration, and invasion. In addition, RT-qPCR and Western Blots showed that the downregulation of circPTPN22 could inhibit the EMT process. In vivo experiments also showed that the downregulation of circPTPN22 could inhibit the growth and EMT process of GC in vivo. Although the regulatory role of circRNA in the occurrence and development of GC has not been fully described, many reports have found that circRNA can act as ceRNA binding miRNA [21, 22]. Our study predicted that circPTPN22 connected many miRNAs through the prediction website, and the has_miR_665 with the highest score was selected after combining with circInteractome. Previous studies have shown that has_miR_665 is down-regulated in GC and could significantly inhibit the proliferation, invasion, and EMT process of GC cells by targeting PPP2R2A. In conclusion, circPTPN22 may regulate GC through the ceRNA mechanism.

## Conclusion

CircPTPN22 was selected by circRNA sequencing, and RT-qPCR results showed that circPTPN22 is up-regulated in GC tissues, cells, and plasma. According to the experimental results, circPTPN22 can be an effective diagnostic and prognostic biomarker. In vitro and vivo experiments showed that down-regulation of circPTPN22 expression could inhibit the proliferation and metastasis of GC cells by affecting the EMT pathway, which may, through the ceRNA mechanism, influence the progress of the GC.

## Supplementary Information

**Additional file 1: Table S1** The CV of intra assay and inter assay.

**Additional file 2: Fig. S1** Feasibility assessment of circPTPN22 detected by RT-QPCR.

**Additional file 3: Fig. S2** Transfection efficiency of sh-control,sh-1 and sh-2 in MKN-1 and AGS cells.

## Data Availability

All datasets presented in this study are included in the article/additional files.

## Abbreviations

GC: Gastric cancer
CircRNAs: Circular RNAs
RT-qPCR: Quantitative reverse transcription PCR
EMT: Epithelial-mesenchymal transformation
E-cad: E-cadherin
N-cad: N-cadherin
mRNA: Messenger RNA
FBS: Fetal bovine serum
RNase R: Ribonuclease R
SPSS: Statistical Product and Service Solutions
miRNA: MicroRNA
cDNA: Complementary DNA
ROC: Receiver operating characteristic
CEA: Carcinoembryonic antigen
CA199: Carbohydrate antigen199
ncRNAs: Non-coding RNAs
