# Supplementary figures and images for: As a biomarker for gastric cancer, circPTPN22 regulates the progression of gastric cancer through the EMT pathway
Source: Cancer Cell Int. 2021 Jan 11;21:44. doi: 10.1186/s12935-020-01701-1 (PMC7802183; doi:10.1186/s12935-020-01701-1)

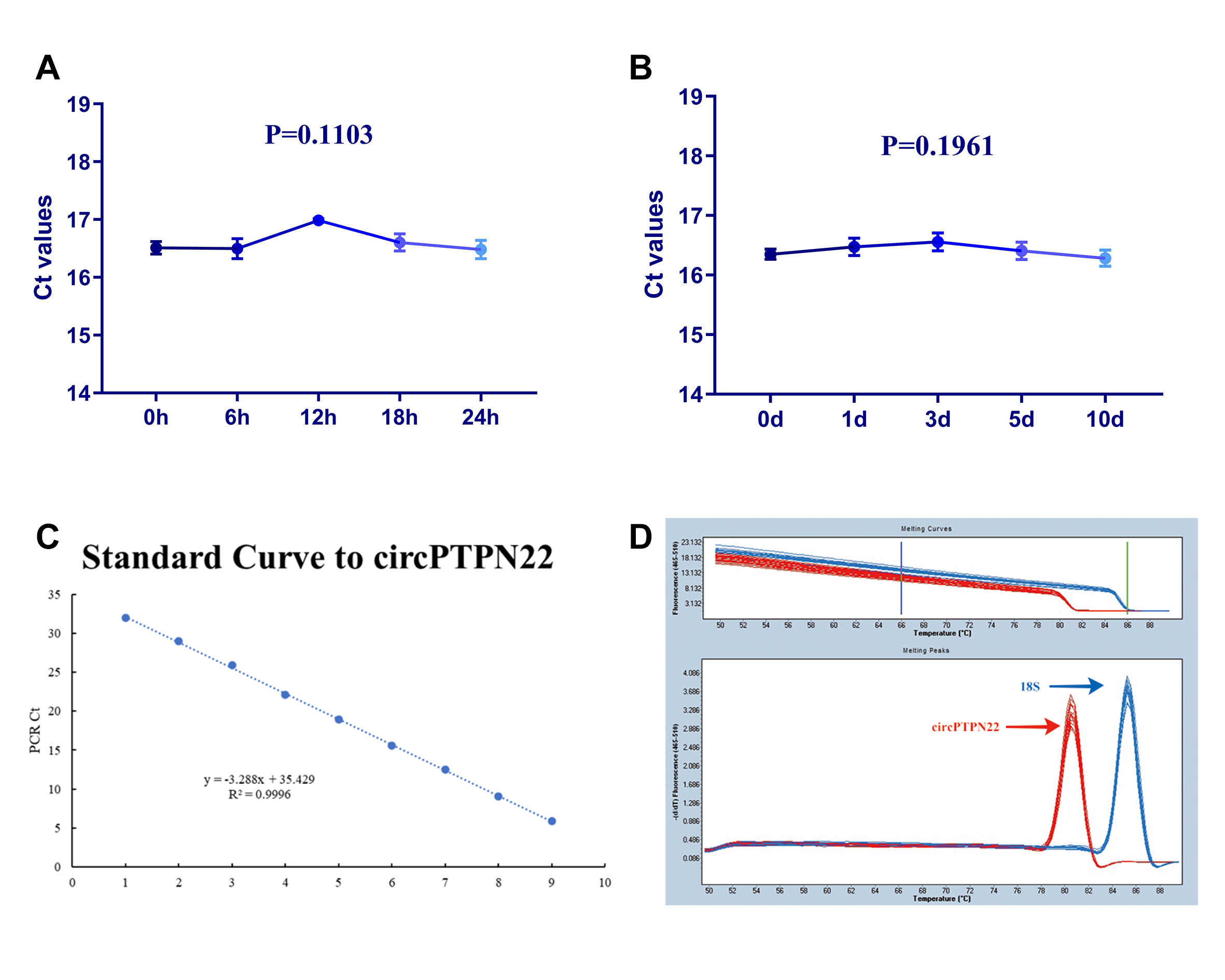

Supplement: Supplementary file 2 — Additional file 2: Fig. S1 Feasibility assessment of circPTPN22 detected by RT-QPCR. [file 12935_2020_1701_MOESM2_ESM.tif]

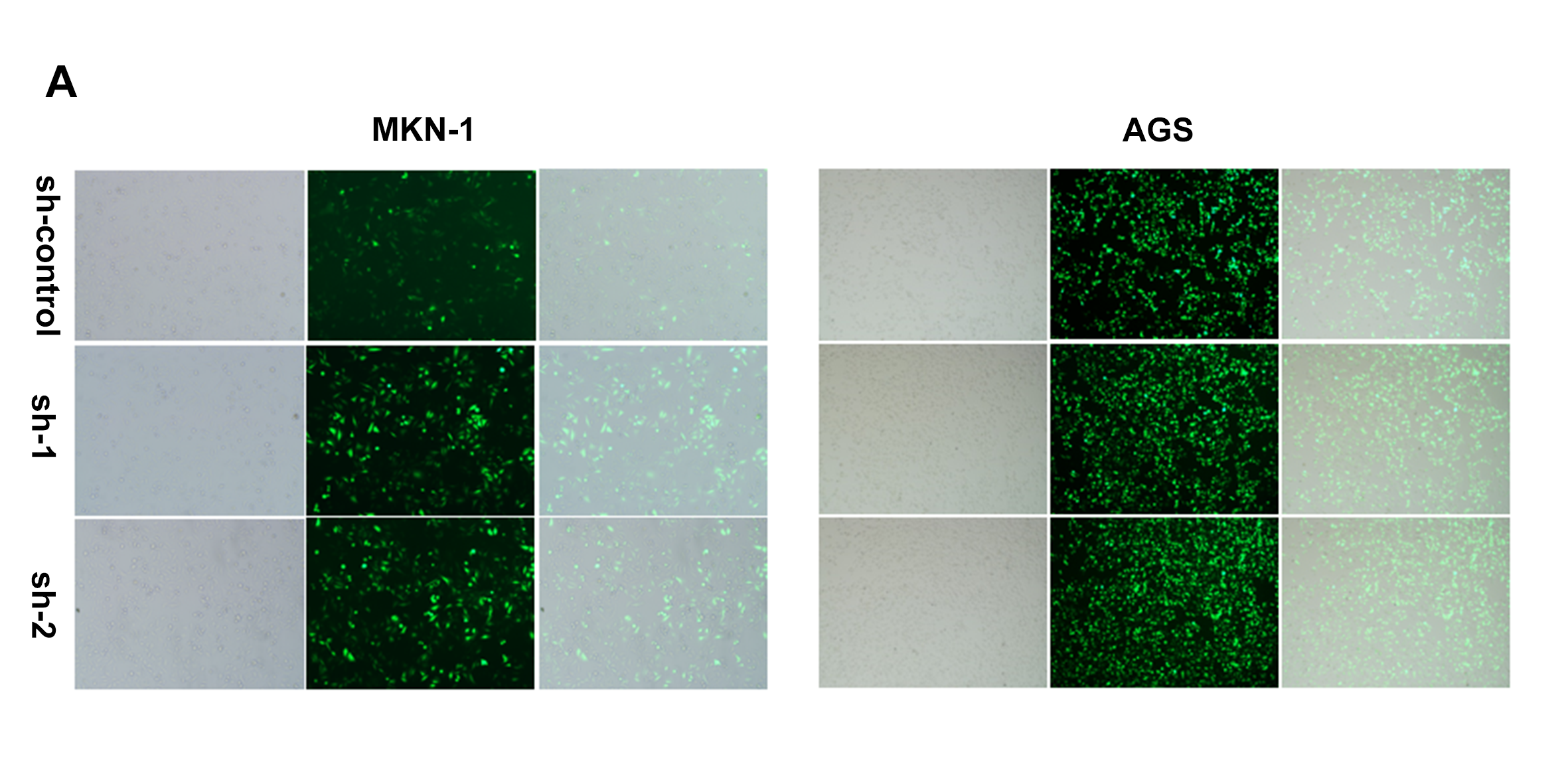

Supplement: Supplementary file 3 — Additional file 3: Fig. S2 Transfection efficiency of sh-control,sh-1 and sh-2 in MKN-1 and AGS cells. [file 12935_2020_1701_MOESM3_ESM.tif]
